# Indigenous perspectives on active living in remote Australia: a qualitative exploration of the socio-cultural link between health, the environment and economics

**DOI:** 10.1186/1471-2458-13-473

**Published:** 2013-05-15

**Authors:** Sharon L Thompson, Richard D Chenhall, Julie K Brimblecombe

**Affiliations:** 1 Menzies School of Health Research; Institute of Advanced Studies, Charles Darwin University, PO Box 41096, Casuarina, Northern Territory 0811, Australia; 2 Melbourne School of Population and Global Health, University of Melbourne, 207 Bouverie St, Carlton, Victoria, 3010, Australia

**Keywords:** Indigenous, Chronic disease, Physical activity, Active living, Indigenous natural and cultural resource management, Remote Australia

## Abstract

**Background:**

The burden of chronic disease in Indigenous Australia is more than double that of non-Indigenous populations and even higher in remote Northern Territory (NT) communities. Sufficient levels of physical activity are known to reduce the risk of chronic disease and improve the health of those already suffering from chronic disease. It has been identified that effective promotion of physical activity in Indigenous settings requires the diverse cultural perspectives and participation of Indigenous people. However, Indigenous concepts of physical activity are not represented in the public health literature and examples of Indigenous involvement in physical activity promotion are scarce. This study aimed to explore and describe local perspectives, experiences and meanings of physical activity in two remote NT Indigenous communities.

**Methods:**

Qualitative research methods guided by ethnographic and participatory action research principles were used. Semi-structured interviews conducted with 23 purposively selected community members were the main source of data, augmented by five commissioned paintings by community-based artists and observations recorded in a journal by the first author.

**Results:**

The findings reveal that in this cultural context the meaning of physical activity is embedded in socially significant and economically necessary physical engagement with the environment. Participants described physical activities associated with Indigenous natural and cultural resource management, customary spaces, seasonal timing and traditional education as creating and protecting health. These activities were viewed not only as culturally appropriate physical activities that contribute to health but as legitimate, physically active forms of social organisation, education and employment that help to build and maintain relationships, wealth, resources and the environment.

**Conclusion:**

This different construction of physical activity in remote Indigenous communities highlights the importance of involving Indigenous people in the development and implementation of physical activity promotion. Physical activities associated with traditional Indigenous cultural practices and being active ‘on country’ need to be viewed as legitimate health promotion activities. Exploring further ways to enable Indigenous people in remote NT to be involved in creating viable active livelihoods on ‘traditional country’ needs to be considered as imperative to health improvement.

## Background

Growing patterns of chronic disease have emerged in all populations that have adopted modern Western sedentary lifestyles and there is evidence that sufficient levels of physical activity can help prevent, manage and treat these diseases [1]. There is however, little understanding of how physical activity can be successfully reintegrated into daily lives in a rapidly changing environment characterised by labour saving technology, low physical energy requirements and the ready availability of energy dense convenience foods. Historically, health initiatives in Western developed countries have generally attempted to address the negative impacts of sedentarism by targeting individual behaviours through general practice interventions, social marketing campaigns and public health policy. Western biomedical models have generally conceptualised physical activity as a separate health related exercise requiring a minimum of thirty minutes of moderate activity to be inserted into an individual’s routine on most days of the week. As the complexity of the determinants of physical activity emerge and there is acknowledgement that the driving forces lie mostly beyond the control of the individual and outside the health sector; social, environmental and economic factors are receiving more attention [2]. The creation of ‘active living’ communities through multi-level, environmental interventions and policy has more recently been recognised as a promising strategy for urban and suburban settings [3]. To be relevant, meaningful and more likely to be effective in specific populations these strategies need to consider and understand social influences and the different ways various cultures conceptualise activities and health [3,4].

Nationally, Indigenous Australians suffer a disproportionate burden of chronic disease compared to non-Indigenous Australians. Rates of circulatory disease are ten times those of the non-Indigenous population [5]. Obesity and physical inactivity are among the leading contributors to this burden [6]. It is estimated that in *remote* Northern Territory (NT) communities 77% of the 17 year difference in life expectancy between Indigenous and non-Indigenous populations is due to preventable chronic disease [7]. As little as fifty years ago, these remote Indigenous NT societies were subsistence-based and lived in an environment that required maximum energy expenditure for minimal energy density intake and had to conserve physical energy to survive [8]. Although there is no accurate information about current physical activity levels in remote Indigenous populations, the recent and sudden exposure to a sedentary lifestyle and the accompanying elevated burden of diabetes and obesity, suggest that being physically active is possibly even more important in the prevention of chronic disease in this population than it is amongst others [9]. However, the drastically lower levels of health and well-being in the Indigenous population are also associated with a range of social factors stemming from colonisation such as: racism, dispossession, social isolation, poverty, inadequate housing, unemployment and lack of appropriate services, as well as higher rates of smoking, alcohol abuse, poor diet and inactivity [7,9]. Similar health and social issues are experienced by colonised Indigenous people world-wide [10].

There are only a few examples of documented physical activity related initiatives in remote Indigenous communities and little evaluation of their outcomes to learn from [2,9]. The success of Indigenous health promotion strategies however, is known to be influenced by culturally specific perceptions of health and the social norms arising from them [11-13]. The available literature highlights the importance of making links between Indigenous culture and physical activity for initiatives to be effective in Indigenous settings and that these links involve “innovative ways of combining approaches to improve cultural pride, cultural identity and self-esteem with physical activity promotion” [9], p.40]. The literature also suggests that the ineffectiveness of interventions to modify high risk behaviours in Indigenous populations is likely to be due to a lack of understanding of the wider sets of social meanings attached to those behaviours and of the everyday lives of Indigenous Australians [14,15]. Cardiovascular health and lifestyle programs have also been shown to be more successful and sustainable when they involve Indigenous community members, local knowledge and leadership [16-19].

Overall, the need to work in partnership and to establish cultural links with physical activity has been shown to be important in Indigenous contexts but there is an absence of *how* these links and partnerships are made from an Indigenous perspective. Collaborating to identify the Indigenous meanings and lived experience of physical activity in a remote context has been acknowledged as a priority in health promotion. This research aimed to provide some perspectives on physical activity as seen by Indigenous people living remotely in the NT.

## Methods

### Study design

This qualitative study drew upon phenomenological ethnography and was guided by participatory action research and community development principles. Community-based co-researchers were employed and worked together with the first author (a Darwin based migrant Australian of British descent), in the design, facilitation and implementation of the field work.

### Study population and study sites

There are approximately 82 remote communities in the NT. These are spread out over an area of approximately 1,400,000 square kilometres. Recently these communities were grouped into 8 regional shires. An email seeking expressions of interest about participating in the research project was initially sent to all NT regional shire managers, all community clinics and as many community council offices and organisations as possible. Representatives from approximately 7 communities responded and were followed up with phone calls or visits to ascertain the level of interest. During a period of closer consultation with two of the remote community areas the people expressed concern about chronic disease and physical inactivity in their community and saw the research project as an opportunity to improve the situation. An agreement to participate in the project was established and support letters were signed by representatives of the community councils, organisations and traditional owners.

Situated nearly 2000 km apart, one study site is in the tropical Top End and the other in the arid Central Desert region of southern NT. The Top End research setting has an estimated Indigenous population of 1300 and is approximately 400 km from the nearest town. The Central Desert setting has an estimated population of around 800 people and is approximately 800 km from the nearest town. Both communities are isolated and only accessible by light plane for months at a time every wet season. English is a third or fourth language to both groups. Five Bininj (the people of the top end community) were employed as community-based co-researchers in the Top End community. Attempts to recruit Yapa (the people of the desert community) co-researchers in the Central Desert community were unsuccessful. This was mainly due to intra-community conflict and unrest and the subsequent temporary departure of many people from the community preceding and during the field work period. Yapa and non-Indigenous service providers assisted with recruitment and facilitation of interviews in this community.

Like most remote NT communities these settlements have a unique history, with a population made up of traditional owners and combinations of various clan and language groups who are not all originally from that region. Extensive and complex Indigenous legal systems, ancestral connections, kinship structures and skin groups (moieties) define identities, relationships, roles, social standing and power [20]. Various traditional and contemporary laws, customs, beliefs and spiritual traditions together with those brought in by Europeans and technology are woven into the fabric of everyday life in the settlements.

Typical to most community settlements, those in this study cover a small geographical area and are surrounded by vast expanses of undeveloped vegetation and wildlife. They feature: very basic and often inadequate infrastructure and services, including a council office, school, clinic, an aged care facility and shop; mostly dirt and some sealed roads; overcrowded houses; running water; power (often supplied by diesel generators or solar systems); some shade structures; a small number of cars; a basic outdoor basketball court; football ground; playground and recently in a few communities - a pool.

### Data collection and analysis

Purposive and opportunistic methods were used to recruit participants over a five month period with the aim of interviewing at least 30 community members or until data saturation. We sought a balance of genders and a range of ages over 16 in our study group. Available male and female community members and co-researchers over the age of 16 were invited in person by the researchers or service providers to be interviewed.

Semi-structured, in-depth interviews were held in informal locations chosen or deemed suitable by co-researchers, participants or service providers. Trialling unstructured interviews enabled broad guiding topics to be formulated which were then used to semi-structure the discussions. Interviews were recorded with informed consent using digital voice recorders. Those held in local language were interpreted by bilingual co-researchers, although most were held in English. Five of the participants contributed commissioned paintings representing perspectives and meanings of physical activity. Observations of community infrastructure, physical activity and social and cultural events were recorded in a journal by the first author.

Interviews were transcribed externally and reviewed for accuracy. Thematic analysis was used to organise and interpret the material. Persistent themes recurring across the interviews and paintings were identified, grouped and contextualised in relation to each other and to current social, cultural, Indigenous and public health literature. The interpretation and findings were compiled into a booklet and poster using quotes and images of the themes. These visual mediums were used by the co-researchers and available participants to read over, reflect upon and communicate with the first researcher about the results. In this way the thematic interpretation of the data was discussed and then confirmed as representative of the views of those involved in the study.

### Ethical approval

The study received ethics clearance through the Human Research Ethics Committees of both Northern and Central Australia. Ethical issues related to this project involved inadvertent researcher dominance, inappropriateness or disrespect as a result of lack of awareness of and insensitivity to, cultural differences. The possibility of this occurring was limited as much as possible by adhering to the Cultural Respect Framework and the principles for ethical conduct in Aboriginal and Torres Strait Islander Research as outlined by the National Health and Medical Research Council (NHMRC) - reciprocity, respect, equality, responsibility, survival and protection and spirit and integrity [21]. The project was also informed by the NHMRC Road Map priorities, principles and processes, and tried to ensure: a holistic view of health; community involvement in the development and conduct of the research; enhancing skill development around research methods and health promotion by employing community-based research assistants; and producing practical outcomes relevant to Indigenous people and their service providers [22]. The researcher sought guidance from community-based Indigenous organisations, community members, community-based research assistants and traditional owners regarding matters of cultural protocol and sensitivity, and attempted to maintain continued awareness of issues around; communication style differences; language barriers; informed consent and appropriate feedback.

## Results

All participants from the Top End community (number =17, male = 9, female =8) were aged between 26 and 65 years. Due to time and distance limitations, community issues and staff recruitment difficulties in the Central Desert, only females (number =6) already involved in some form of organised physical activity were recruited. This included two younger participants aged between 16 and 25 years. The total number of respondents was 23.

Findings from the study were fed back to community members, participants and stakeholders. In this paper the findings are contextualised by some observations from the journal of the first author and organised around three key themes: i) the economic meanings of physical activity; ii) the social organisation of physical activity; and iii) physical activity on traditional country. Participants requested to be referred to as Bininj or Yapa and individuals have been given pseudonyms.

### Setting the scene: some observations

Even with the presence of cars and the heat in tropical and desert communities, people of all ages often go about their business on foot in small groups, mainly in cooler parts of the day, at a slow pace, walking on the roads and well worn dirt paths. Despite the service providers who participated in this project reporting a preference by community members for travel by car even over short distances, the lack of supply and maintenance of cars and licenses, and the cost of fuel, means that many people also walk when getting around in the community. At various times throughout the year residents go “out bush”, into the nearby and vast areas outside the community to collect resources, hunt food, participate in ceremony, swim, or visit traditional country, other communities and distant towns.

Team sports like football, basketball and softball are popular among the young and small league competitions are found in many remote communities. However, it is hard to find examples of ongoing programs capable of increasing activity levels of the majority of adults who do not play sport and the many at risk of developing or already suffering from chronic disease. The visible physical activity promotion that does occur in these communities happens indirectly through school sports and annual community festival activities; and directly through visiting health professionals, and where available, community-based public health staff [23,24]. These are often short term, one-off or irregular programs driven by people from outside the community or by those who only stay a short time and thereby are often based on non-Indigenous cultural perceptions of physical activity and health [23].

### Work and ‘walkabout’: the economic meanings of physical activity

During interviews it was apparent that engaging in physical activity just to improve health did not factor highly if at all, in the day-to-day lives of the participants. Rather for most Bininj and Yapa participants there were strong associations between physical activity, the land and work. Some Bininj men even defined the meaning of ‘physical activity’ as most closely related to that of the Kunwinjku word ‘*durrkmirri’;* meaning, ‘*to work’*.

When describing physical activities participants referred mainly in their stories to those with specific purpose related to the consumption and distribution of natural and cultural resources including the acquisition of food, collecting wood for fires, fibres and dyes for weaving and painting, the building of shelters and the passing on of knowledge through walking, ceremony and dancing. Walking was mainly described by Bininj and Yapa women as an essential activity with many purposes*.*

Lorene: …when you are walking out there, you are looking around for things as well & digging things up & grabbing things off trees. (Yapa woman, 26-35).

Cynthia: Walking and doing something at the same time. Like looking and digging. Us women we love to hunt. (Yapa woman, 46+)

Groups of significant others, usually family, were always involved in the activities. This relationship between physical activity, work and family can be seen in the comments of Yapa woman, Lorene:

Lorene: Well, when I go, we go as a family and I take the kids and we go out for a walk, digging for witchetty grubs & picking up wood … walking around the bush … mainly on weekends … Like whatever (bush foods) you get, you have got someone there making fire or digging the hole ready, getting fire wood & getting other little pieces & getting leaves for the (food) to be cut on. (Yapa woman, 25-36)

Even with the influence of the paid working week and shops these traditional food-related work practices continued in the lives of the participants.

Ruby: At the shop they always sell buffalo meat, that’s all, and bullock meat. But when we go out during weekends we can get magpie geese, turtle, file snake, or pig, fresh ones, or flying fox, and (the) other thing is, we can get mud mussel from the creek and just boil it! … Out in the bush we can get yabbies, prawn … red claw … Sometimes we go for a swim, drag the net, get everything - all sorts of fish! (Bininj woman, 26-35)

In the increasingly sedentary setting of the remote community, the responses of Bininj and Yapa men and women demonstrated that, where possible, they were attempting to maintain subsistence-based, physical work activities on weekends.

Campbell: Well, we do fishing (and) hunting, but that’s only for one day, maybe two days. Just on weekends; Saturday and Sunday.” (Bininj man, 36-45).

*Nancy: Probably just on Saturdays. Take (all the) ladies, go for a walk, Look for witchetty grubs.* (*Yapa woman, 36-45)*

Consistent with the literature, participants described how wealth exists not only in the natural resources themselves but in people’s connection to those resources which are demonstrated through their presence, interaction and the passing down of knowledge [25,26]. The physical process of tending to the renewal and reproduction of these resources, the manner of collection or hunting of food, building of shelters from local materials, preparing bark for painting or pandanus for weaving or dancing in ceremony, involves physical activity but is also valuable work performed for the benefit of the group rather than for the individual.

A painting created by a female participant from the Central Desert with the intention of expressing and promoting physical activity shows food sources being collected by four women and brought together to eat and share in a space in the centre of the painting. Footprints depict how walking has been utilised not only to collect the food from each place but also plays a part in sharing what has been collected.

Another interpretation of healthy physical activity portrayed in a painting by one of the Top End male participants called ‘Bininj erecting a bark shelter’, is an example of an Indigenous activity which is likely to go unrecognised as physically active work. As is shown in the painting, creating dwellings according to cultural and environmental necessity is for this participant a legitimate, health promoting activity. Examples of some of these kinds of work becoming recognised and a form of paid employment are found in some communities in limited supply in the form of land management and youth diversion programs. The management work involves native vegetation regeneration, weed, feral animal, water and fire control and the youth activities involve hunting, camping and walking on traditional country with elders. Eugene*,* a male participant working as a ranger to manage natural resources in the Top End community, described how this kind of work allowed him to meet his traditional physical activity and dietary needs, and the needs of the environment, in a culturally appropriate way.

Eugene: Well I walk a lot. You know how you see on T.V, you know, people riding and walking and exercising? Well… you know, we (are) working (as rangers) and exercising at the same time. I mean when you go out there (in the bush), you know, we have kangaroo or goose for (a) feed … we’ll (remove) weeds (at the same time), we walk like five kms or twenty kms, go (a) long way … and we carry that (heavy) spray pack. Well that’s mainly what we do see? Constantly walking and that’s our exercise for the whole day (Bininj man, 36-45).

Similarly, Dan described how living and working on one’s own country contributes to Bininj economic and health needs:

Dan: Like in another man(s) country (you) might be poor but when you go back home – you’ll be rich! You’ve got own land see? “I want (those who are homeless in town) to come back home and live a better life so we can see (them) and make a longer life. (Bininj man, 26-35).

To participants, ‘the bush’ was not considered a remote, inhospitable place, it was home; a place where these physically active forms of mostly unpaid work and environmental management were essential and provided a reliable source of food, security, social cohesion and pride.

### Activity, time and place: the social organisation of physical activities

Participant responses highlighted different cultural preferences for the way physical activities are constructed and organised, the spaces they take place in and the times they occur. Many said they and other adults did not and would not utilise physical activity programs or spaces, such as swimming pools, youth centres, basketball courts or walking groups. The comments of the Bininj women reflected a willingness and preference to ‘do things (their) own way, in the bush’ rather than the ‘Balanda (European) way’. Swimming in a billabong was seen to be more culturally appropriate than swimming in a public pool. Bininj and Yapa women’s comments exemplified how for them to be active or to be seen to be active, in public pools or jogging, was viewed as culturally unacceptable. For example:

Would people come to exercise in a pool?

Alice: Well I think I don’t like (doing this) … It’s different like, other people might think “oh these black people don’t usually do that swimming around & exercising, running or jogging … (Bininj woman, 36-45)

And Bininj elder Sadie explains with humour the unsuitability of jogging in her outstation home.

What about that Balanda (European) exercise, like you know, you see Balanda go … running in the afternoon or things like that. What do you think of that?

*Sadie: I tell you what; I wouldn’t run!* (Laughing*). Bloody buffalo make me run, that’s all. I’ll be moving all right!* (All laughing). *Yeah, (we) just walk, walk. (Bininj Woman, 36-45)*

European forms of physical activity, facilities and resources were also often seen as being suitable for children only.

Do you think people like doing Balanda exercise?

Eugene: (They) wouldn’t (like it). I think … the kids would be interested, but with adults, I doubt it. Like, you could try anyway. Yeah. You could ride a bike around here but … no, only kids ride bikes around here. (Bininj Man, 36-45).

‘Fitness’ from a sociological perspective has been defined as “a relationship between a person’s psychomotor capacities and … the socio-material organisation of space, time and activity. In this context, fitness is a secure and comfortable relationship with a socially organised and materially constructed environment” [27]. The material construction of the community setting was described by most participants negatively, as uncomfortable and not lending itself to ‘fitness’ in this sense. People described themselves as feeling more comfortable and able to be active in their own cultural spaces, in private and in accordance with their own purposes and seasonal time structures. Physical activities carried out in ‘the bush’ were described as different to those carried out in the community according to the nature of the environment and the comfort of the individual’s relationship with it.

Campbell: (Sam, a Balanda man) likes to run, he likes the exercise. Yeah. Funny thing (though), out in the bush it’s not worth it. Because it is hot, you get dehydrated quick(ly) & you feel dizzy, (and) all that. (Sam) was saying, ’Stay healthy, stay fit’ (in the community) but out there (in the bush) he was crying! And my cousin, he’s short, fat & unfit, not really fat (but) solid … (and) he was fine walking (in the bush). (Bininj man, 36-45)

Campbell also described an experience and observation of the differences between his comfortable and secure way of walking quickly in the bush compared to the ‘slow and steady’ way of walking of someone not familiar with their surrounds.

Campbell: (When I was walking in the bush) I was just walking and climbing and walking … walking after dark. And she (Balanda) was walking slowly, steady, in her step over the rocks. And she ask(ed) me “how (did) you manage to walk like that the whole (time)?” (I said), “I grew up with it. My legs (have) got all the balance. I can run, I also run fast on the rocks. I don’t fall down. If I’m walking out (there and) there’s no sugar bags (native bees hive), I know which grass I can eat that (has) got sugar in it (to keep me going). (Bininj man, 36-45)

Appropriate times to be physically active were seen mainly as part of an existing relationship between place, time, people and activities as determined by the seasons, the sun, weather patterns, environmental conditions and the availability of food sources. Comments about the role of seasonal timing and the availability of certain foods in the organisation of physical activities support previous observations that in Indigenous Australian cultures human activities are traditionally seen to be primarily designated by season and location (Broome in [20]. Additionally, the availability of a vehicle and fuel to reach those locations was also considered necessary and appropriate.

To illustrate the importance of the relationship between activity, place and time, Bininj women and men described when they go looking for yam or goose in this way:

How much do you still go collecting yam or digging yam …?

Alice: Ground is soft for yam at end of wet season. That’s when it’s soft. Now it’s really dry and you can’t dig. (Bininj woman, 36-45).

Dan: Like if you (are) coming (out of the) wet season you can catch goose and you (have) got (those) bush plum (too) …. (Bininj man, 26-35)

Notably, it was only those already diagnosed with chronic disease or obesity (‘Balanda sicknesses’) that considered participating in, or were considered likely to participate in, ‘Balanda’ or ‘Kardiya’ (Central Desert word for non-Indigenous) exercise programs like a walking group:

Why do you want to go walking in a walking group?

Nancy: I want to lose weight. Also I am a diabetic too. (Yapa woman, 36-45)

Mimi: My mum and my other aunty they (were) talking about walking around because they are really big you know? Because they are diabetic as well, they have got big stomachs. (Yapa young woman, 16-25).

### Being ‘on country’: physical activity on traditional country

When it comes to physical activity and health we can see from the interpretation of the perceptions, experiences and meanings expressed by participants in these themes why interaction with the natural environment is important. Natural and cultural resource management and associated activities that occur ‘on country’ were identified as the preferred health promoting physical activities. This last theme reveals how being on country is an important part of re-establishing physical activity as part of everyday life in a remote community and presents organised outdoor programs as a strategy to support this connection.

Both male and female, Bininj and Yapa participants often talked about traditional country or ‘the bush’ as an active healthy place and the community as an inactive, unhealthy place.

Marcia: When he came in … he said something about going bush and I said, “oh, that’s good, I’m glad you’re going out bush, get away … And I said, “I don’t want to go visiting you on ‘the machine’ (dialysis). You want to be on that machine or what? … and he cracked up laughing and said, “no, that’s why I’m going bush – I just want to get away from the temptations …(Bininj woman, 46+)

Activities on country were also viewed as an educational opportunity that could facilitate knowledge transfer about how to find bush foods and survive in ‘the bush’.

Noel: That’s teaching them - walking the kids across the land. (That) teaches them what’s good for them … and that’s about physical exercise that we(‘re) talking about … I’ve experienced living off the land; out in Central Arnhem Land. That’s it! It was good. People used to walk miles and collect their food…and that’s healthy living and they would live in that (healthy way) … Yeah, I mean that (bush walking) is addressing those bush tucker (issues), and teaching them how to find that bush tucker … Those (are the) sort of experiences that they (health promoters) should be looking at because that’s part of it, it will (make) that healthy mind (know how) to find a healthy ‘manme’ (local word for food). Proper one, right one! (Bininj man, 36-45)

Both male and female, Bininj and Yapa recounted taking part in annual organised bush walks on country that allowed them to fulfil their cultural obligations to their ancestors and their country. Ruby described the necessity of these connections:

Ruby: That’s why we have to take them (kids, bush walking) so the old people can show them the country and the names of the place. They need to talk about and learn about the name of the places and who belongs to that place. That’s why myself and (my husband) always join in for the bush walks so when we go there they always tell us, “well this is your great, great, great, grandmother country, or grandfather. Like when we stayed there, (at the) first camp, we told (them) “this place here you call your grandfather country …” (Bininj woman, 26-35)

One of the paintings prepared by a male participant shows traditional dancing on country as a form of healthy physical activity. In this image, two Bininj men are participating in a dance as part of a traditional ceremony. The figure on the left is an old man holding clap sticks and covered in ceremonial body painting designs. The figure on the right is a young boy learning from his elder, copying and learning the story of his movements, so that in the future he can pass them on to the next generation. The artist explained that his ancestors were taught to dance by mimi spirits and that dancing was still an important and enjoyable part of his culture today. Walking and being on country in this way is described as a necessity undertaken for a particular meaningful social purpose.

What is the best way to spread the word about why working your body … doing exercise (and) being fit is good?

Campbell: I think it’s best for us (to) like take people out for a bush walk. It’s the best (way) to tell them and also to make them understand what life risks are. I think that bush walking is it! It’s great! (It is) talking (about) everything about life … (Bininj man, 36-45).

Participants looked to their cultural traditions and activities associated with maintaining their heritage as a way forward in overcoming social, cultural, economic and health disadvantages. A desire and willingness to conduct and lead these kinds of activities was expressed by a number of male and female, Bininj and Yapa.

Dan: Like for me I am thinking I don’t want all (these community problems) to happen in the future, I reckon for me, (I want) to make (a) program, an education program – we want to do camping … Get axe, firewood, billy can – like that. We are going to stay there (out bush). That’s how I’m looking at it. Make music … bush tucker … you learn what’s healthy in the bush. Go hunting or fishing … make spear … That’s how I’m looking at it and that’s how I want to be too. Get them to tell their story maybe … write songs … travel around to other communities … learn other people’s culture … (Bininj man, 26-35)

Naomi: I’d like to take people out bush and show them our way. Show them (Balanda) all that bush tucker, you know? We talk about that all the time. (Bininj woman, 36-45)

## Discussion

The meaning of work, space and place in relation to physical activity, as expressed by the participants in this study, suggests that supporting various forms of natural and cultural resource management may be an appropriate physical activity health promotion strategy. It is important that these land management and related employment opportunities involve access to and physical engagement with ‘the bush’ and traditional country.

Meanings and definitions of work have been contested between Indigenous and settler culture since contact. Involved in this contest are diverging perspectives of what constitutes work, leisure, time and payment and how they link together in our relationship to natural resources and the economic environment [23]. Even though Indigenous Australians have sophisticated systems of natural and cultural resource management and work-related activities which continue still and develop where possible in the lives of participants today, Non-Indigenous Australians in general have tended to only recognise ways of working, forms of economics and resource management similar to their own as legitimate [20]. Subsistence activities were often perceived by early settlers as lazy and Indigenous people were reported as being “strongly adverse to labour” (Organ in [20], p.10]). Europeans called these kinds of activity ‘walkabout’; a term still used in the present day and still often associated with laziness and unreliability rather than work [20], p.10]. So despite a long history of a strong working culture unemployment and poverty remain as main determinants of poor Indigenous health in Australia today [8,9].

Even though the inherently physical subsistence activities such as hunting magpie goose or digging for honey ant have not been recognised by non-Indigenous people in general as economically legitimate forms of work, they continue today and have not been totally replaced by Bininj or Yapa with other forms of food production which are likely not to require as much physical activity. The different concepts of work and wealth as explained by the participants are embedded in necessary physical engagement with a particular environment and help explain why on traditional homelands or outstations, where infrastructure and services are few, those who are participating in natural and cultural resource management activities as part of daily life are often the healthiest [14].

Knowing more about how, why, where and when people walk is also an important factor in understanding how to promote physical activity in remote communities. It has been noted by movement specialists that in a cultural context communities consider “uniformity of movement behaviour” as essential for protecting the “stability of the community spirit” (Laban in [28], p. 165]). The notion that physical activity is not a culturally or socially neutral activity is all the more pertinent when viewed from Bourdieu’s concept of *habitus*[29]. *Habitus* is the psychological and physical embodiment of the social and cultural structures in which we live; it is the way the social and cultural world is written into our perceptions, our bodies and actions. The movements required by Western-style physical activity routines imposed on Indigenous people in remote communities thus speaks of a disparity between the non-Indigenous and Indigenous habitus. This has been described by Indigenous academic Bronwyn Fredericks when she stated that Western-style exercise environments “do not necessarily fit within the contexts of the materiality of our bodies” [30].

European style physical activity programs rely upon the Gregorian calendar and the 24 hour clock. The seasonal concepts of time in the tropics and the desert as manifested by different, specific and subtle changes in the environment challenge the unquestioned assumption of the European calendar and clock time as the norm (Adam in [20]). As Glenda Go_djalk, a Yolngu health worker from Marthakal homelands on Elcho Island explains:

You can’t tell the water: “Hey, Stop, stop, stop! Low tide, low tide now! High tide, high tide now! This way! Now that way! It has its own timing for going in, and going out, when it will become rough, and when it will become calm. Yes, this is the same for our bodies [31].

Such cultural differences between Indigenous and non-Indigenous ways of constructing and organising activities, places and times, need to be better understood to promote active living successfully in remote communities. Physical activity programs led by Indigenous people that incorporate the use of customary spaces, seasonal and biological timings are more likely to be meaningful in remote contexts [32,33].

The findings suggest that being and walking on country and regular participation in associated activities are central to the cultivation of Indigenous health in remote areas. These kinds of physical activity allow for the maintenance of social and cultural traditions that are seen to provide a link between health, the environment and economics in this context. Youth diversion and outstation programs in the NT involve facilitating trips to homelands and on to country for people to participate in a range of such activities. As well as increased physical activity, outdoor programs like these have been shown internationally to improve various dimensions of health including well-being, self-efficacy, confidence, independence, peer relations and mental health [26,34,35].

Overall, the participants involved in this study and the themes that they brought up suggest that in a remote Indigenous context being physically active and healthy is about interacting productively and meaningfully with the environment. It is about performing the cultural activities including, but not limited to, resource management and sharing stories about country which provide opportunities for the inter-generational transfer of knowledge and the maintenance of cultural heritage. Internationally, Indigenous people repeatedly associate their extreme ill-health and poverty with their experiences of dispossession and associate solutions with reclaiming access to land [10]. This study shows that in remote Indigenous communities in the NT people refer to being active on traditional country as a solution to many problems deeply embedded in a history of colonisation. These findings support the importance of employment in caring for country and land management programs which enable people to fulfil social and cultural obligations, educate young people, escape the stresses of settlements and earn an income [25] thereby improving the communities health, economics and environment [13,15].

Indigenous cultures have considerable knowledge of human-environment relations and there is growing international evidence to support the promotion of Indigenous cultural and ecological practices to improve health [26,36-38]. Similarly, environmental health and development specialists are recommending that better management of land degradation, biodiversity loss, soil erosion, food insecurity and water quality decline, as well as reducing ‘unsustainable consumption’, “will have long-term and sustained beneficial effects on human health” as a whole [39].

Indigenous subsistence economies and their achievements in sustainable development are beginning to be acknowledged:

Indigenous peoples’ economies now represent the greatest continuity with pre-industrial modes of production and traditional livelihoods in the contemporary world. These economies, representing sustained interaction and adaptation with particular locations and ecosystems, are among the longest-standing and most proven examples of “sustainable development” in the twenty-first century [10].

Recognising the social and cultural dimensions of human-environment relations plays an essential part in the sustainability of human health [38], yet this link between natural and cultural resource management, economics and physical activity in an Indigenous context has only begun to be examined. This connection, as demonstrated through this study, is important in creating what is viewed as ‘a healthy society’, as outlined in the Ottawa Charter of Health Promotion:

the way society organises work should help create a healthy society and … the sustainable use of the natural environment and resources must be addressed in any health promotion strategy [40].

Public health strategists can recognise Indigenous perspectives and capacity and facilitate this connection also by working together with outdoor education and youth diversion programs. Particularly relevant are environmental and socio-ecological outdoor education models that prioritise the importance of human-environment relations [34,35]. These models take a strength-based approach, support activities that are coordinated with ecological rhythms and promote the active use of existing spaces in which people already feel comfortable and secure [32]. They create opportunities for increased physical activity, connection with nature, culture, social networking and Indigenous and non-Indigenous skills transfer [29,30]. For example, an account of an ethno-ecological education camp in Indigenous Canada describes how the Indigenous and non-Indigenous organisers aligned the program and themselves with the physical, emotional, mental and spiritual aspects of the Dene mountain people of Canada’s north, as well as modern science and technology [26]. In this way “the camp brings Indigenous instructors to the forefront as mentors who have something not only useful, but indeed necessary, to teach us” [26]. The participants in this study expressed the need for programs like these to improve physical activity and health.

### Limitations

There are many challenges involved in the process of engaging meaningfully and effectively with people living in remote Australian communities for research purposes due to a difficult history, language and cultural differences, and varying cultural constructions of power, time and priorities. This study was conducted over one year, was only able to explore the views of a limited number of community residents and a majority of those were female in the desert community. The inability to recruit community-based researchers and more participants in the desert community was attributed to a period of intra-community conflict that had preceded the field work and resulted in a significant proportion of the community moving away temporarily. This included 3 people who had initially expressed a strong interest in the research positions moving away from the community during the field work period. This meant that we were not able to document male perspectives or those of less active females in the desert community. However, despite the resulting differences in recruitment methods, participant numbers and the gender imbalance between the groups, many views and experiences described by participants in relation to physical activity were similar within and across the two communities. The men and women, the active and the inactive all described a distinct preference for traditional physical activities in ‘the bush’. The strengths of this study were: the involvement of local co-researchers where possible; the conduct of some interviews in first language; the ability of the co-researchers to translate the local language into English where needed; and the rich contribution made by the paintings of physical activity.

Considering the paucity of literature available, as well the specific challenges of working with remote Indigenous communities, long-term research and interdisciplinary research teams working in partnership with communities are needed to develop effective physical activity health promotion models, guide implementation strategies and achieve substantial benefits.

## Conclusion

These findings help to explain *how* from an Indigenous perspective the meaning of physical activity is connected to culture through work, diet, relationships, place, time and country. This helps to establish some of the socio-cultural links between physical activity, health, the environment and economics in remote northern Indigenous Australia.

Currently situated in a period of unprecedented global economic, environmental and lifestyle disease crises, public health advocates need to recognise Indigenous models of health promotion, resource management and associated contemporary and customary activities as sustainable, healthy systems of active livelihoods [25,41]. This paper demonstrates the necessity of collaborating with Indigenous community members when developing and implementing physical activity programs, as well as the legitimacy of enabling people living remotely to be active on traditional country as a broad inter-sectoral health strategy.

## Competing interests

The authors declare that they have no competing interests.

## Authors’ contributions

All of the authors contributed to the conception and design of the project. JB provided supervision on all aspects of the study. ST and the community-based co-researchers were responsible for data collection and the interpretation of the findings. ST analysed the data and prepared the manuscript; the remaining authors made revisions. All authors read and approved the final manuscript.

## Pre-publication history

The pre-publication history for this paper can be accessed here:

http://www.biomedcentral.com/1471-2458/13/473/prepub
